# Author Correction: Dysfunctional adipocytes promote tumor progression through YAP/TAZ-dependent cancer-associated adipocyte transformation

**DOI:** 10.1038/s41467-024-49720-0

**Published:** 2024-07-04

**Authors:** Yaechan Song, Heeju Na, Seung Eon Lee, You Min Kim, Jihyun Moon, Tae Wook Nam, Yul Ji, Young Jin, Jae Hyung Park, Seok Chan Cho, Jaehoon Lee, Daehee Hwang, Sang-Jun Ha, Hyun Woo Park, Jae Bum Kim, Han-Woong Lee

**Affiliations:** 1 Department of Biochemistry, College of Life Science and Biotechnology, Yonsei University, Seoul, 03722 Republic of Korea; 2 Department of Biological Sciences, Seoul National University, Seoul, 08826 Republic of Korea; 3 Gemcro, Inc, Seoul, 03722 Republic of Korea

**Keywords:** Cancer microenvironment, Breast cancer, Fats, Cancer microenvironment, Breast cancer

Correction to: *Nature Communications* 10.1038/s41467-024-48179-3, published online 14 May 2024

In the acknowledgements section, firstly the funding from National Research Foundation (NRF-2020M3F7A1094089) and Ministry of Science and ICT (RS-2023-00224201) were omitted. Secondly, the program name ‘Brain Korea 21 (BK21) PLUS program’ was written incorrectly and should have read ‘Brain Korea 21 (BK21) Four program’. The original article has been corrected.

