# Supplementary material for: Dysfunctional adipocytes promote tumor progression through YAP/TAZ-dependent cancer-associated adipocyte transformation
Source: Nat Commun. 2024 May 14;15:4052. doi: 10.1038/s41467-024-48179-3 (PMC11094189; doi:10.1038/s41467-024-48179-3)
Supplement: Supplementary file 1 — Supplementary Information [file 41467_2024_48179_MOESM1_ESM.pdf]

Supplementary Information

Dysfunctional Adipocytes Promote Tumor Progression Through YAP/TAZ-dependent Cancer-Associated Adipocyte Transformation

Yaechan Song[1,#], Heeju Na[1,#], Seung Eon Lee[1], You Min Kim[1], Jihyun Moon[1], Tae Wook Nam[1], Yul Ji[2], Young Jin[1], Jae Hyung Park[1], Seok Chan Cho[1], Jaehoon Lee[1,3], Daehee Hwang[2], Sang-Jun Ha[1], Hyun Woo Park[1], Jae Bum Kim[2], Han-Woong Lee[1,3]*

[1] Department of Biochemistry, College of Life Science and Biotechnology, Yonsei University, Seoul 03722, Republic of Korea

[2] Department of Biological Sciences, Seoul National University, Seoul 08826, Republic of Korea

[3] Gemcro, Inc., Seoul 03722, Republic of Korea

*Corresponding author: hwl@yonsei.ac.kr

Index:

Supplementary figures S1 to S11

Supplementary tables S1 to S4

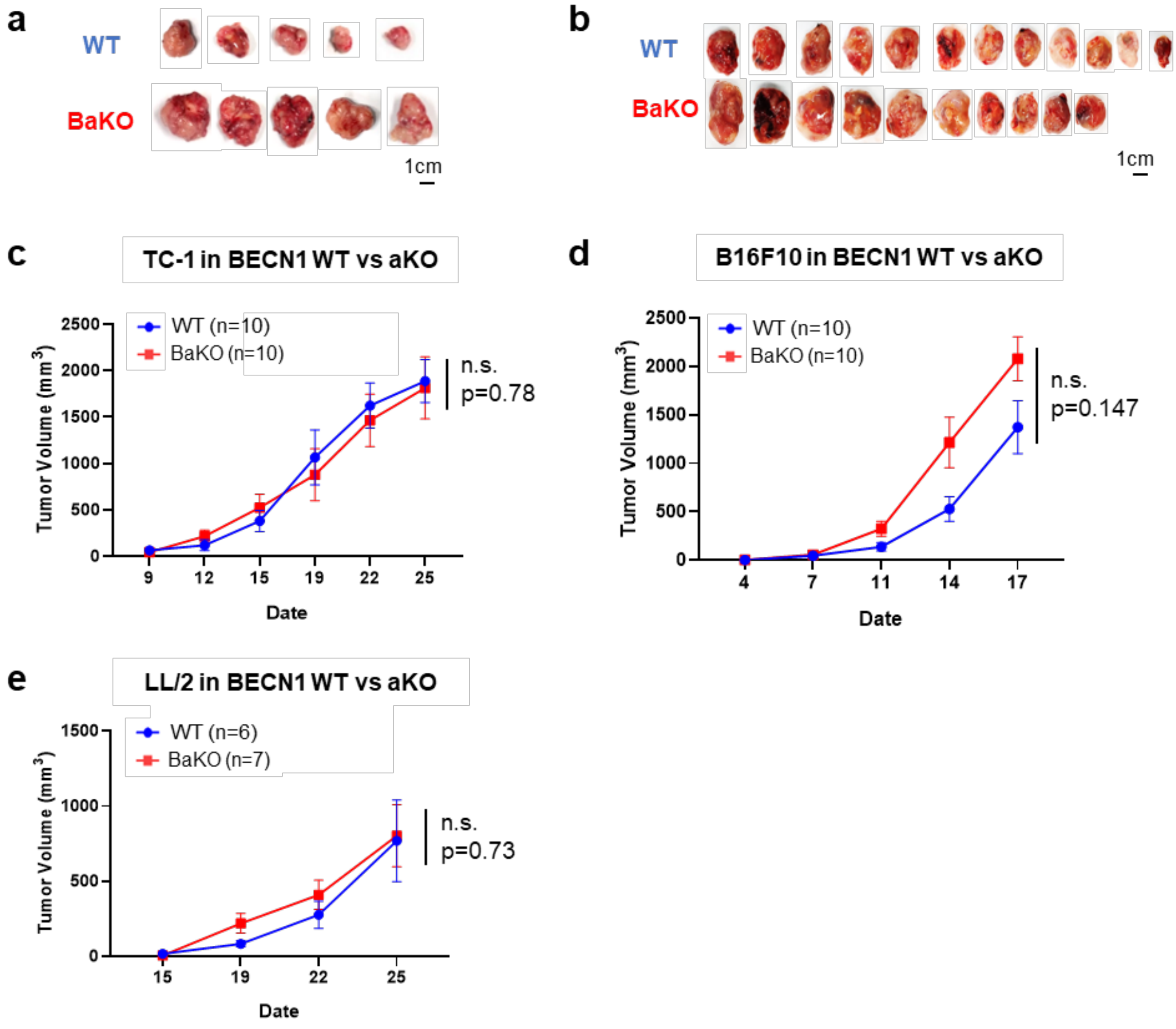

Supplementary Figure 1: Tumor progression of various cancer cell lines in BaKO. (a,b) Representative image of resected tumors presented in Fig. 1a and b, respectively. (c) Growth kinetics after subcutaneous injection of TC1, (d) B16F10, and (e) LL/2 into 8-week-old WT and BaKO mice (sample sizes are as indicated).

Statistics were calculated using ordinary two-way ANOVA (c, d, and e). Data are shown as mean $\pm$ SEM; n.s. = not significant.

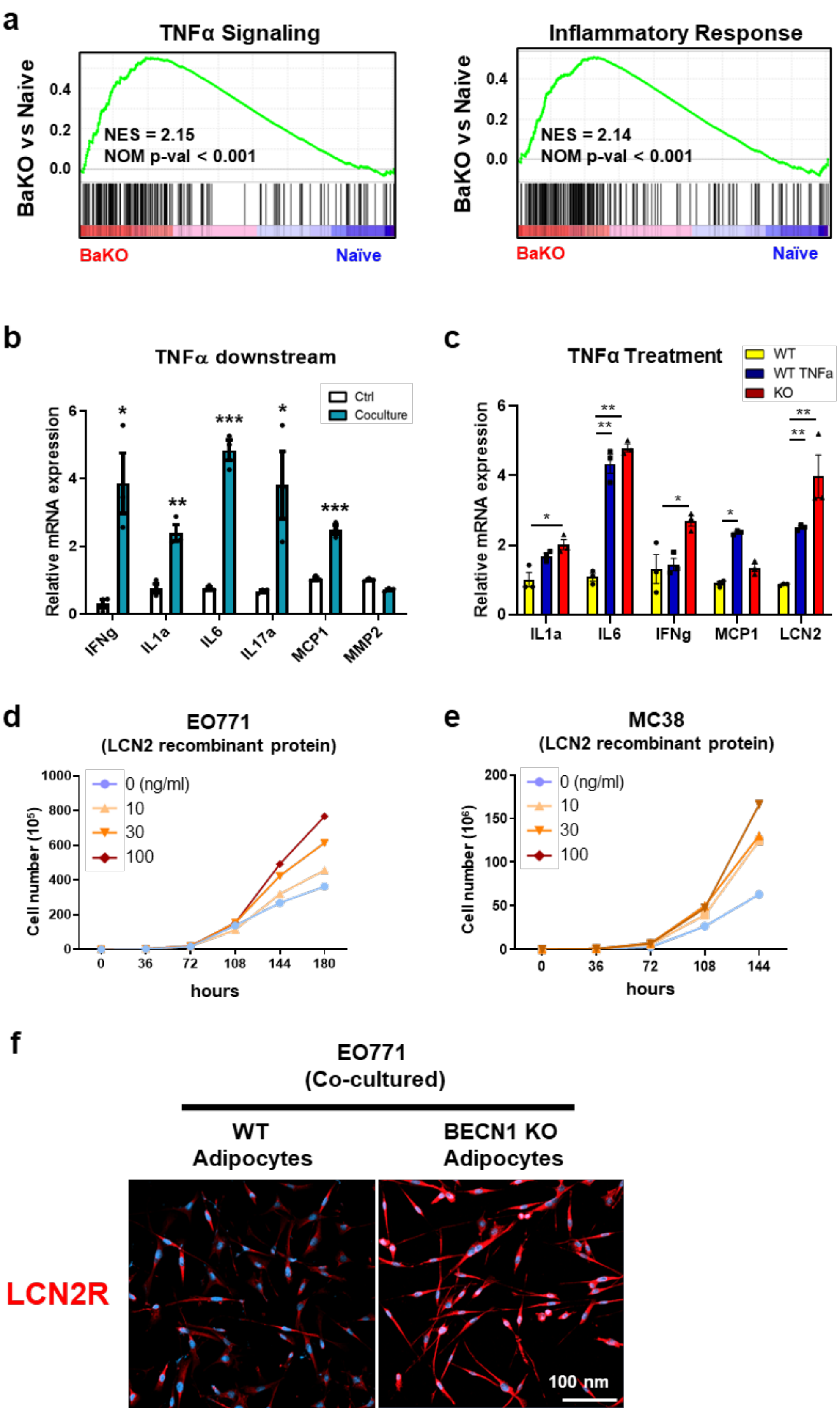

Supplementary Figure 2: TNFα and LCN2 from BECN1 deficient adipocytes support EO771 and MC38 progression. (a) GSEA plots for TNFα signaling and inflammatory response gene sets compared between RNA-seq results of BaKO and naïve WATs. (b) Relative mRNA expression of TNFα downstream genes from control adipocytes and cancer co-cultured adipocytes (n = 3). (c) Relative mRNA expression of TNFα downstream genes and LCN2 after treating adipocytes with or without TNFα recombinant protein (n = 3). (d) Viable cell counting of EO771 and MC-38 (e) treated with LCN2 recombinant protein as dose indicated. Cancer cell numbers were counted as they were sub-cultured every 36 h (n = 2, independent cells per group). (f) Representative IF image of LCN2R in co-cultured EO771 with WT or BECN1 KO adipocytes for 2 days.

Statistics were calculated using two-tailed unpaired students *t*-test (b and c). Data are shown as mean ± SEM; $*p \leq 0.05$, $**p \leq 0.01$, $***p \leq 0.001$.

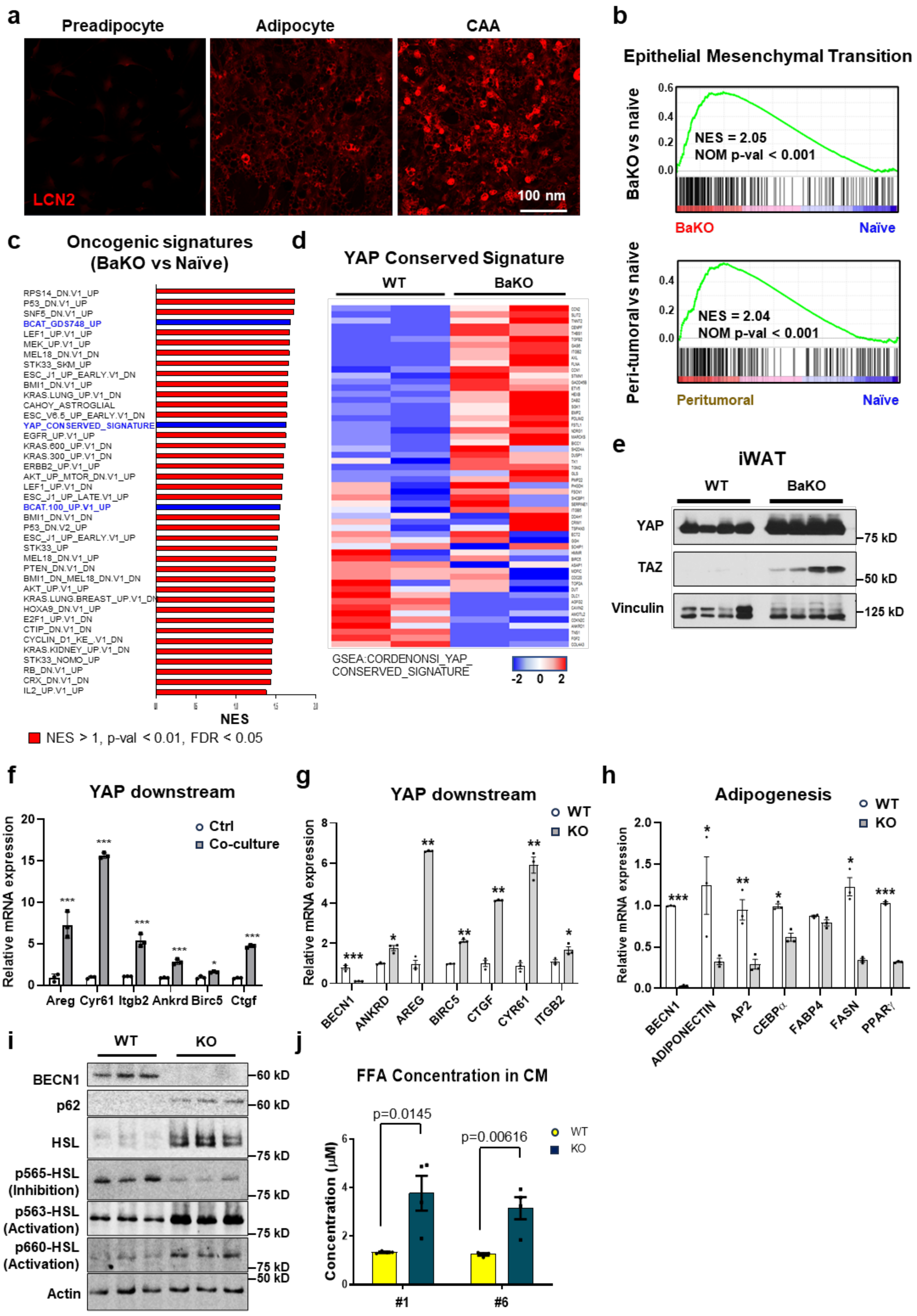
a
Preadipocyte
Adipocyte
CAA
LCN2
100 nm
b
Epithelial Mesenchymal Transition
BaKO vs naive
NES = 2.05
NOM p-val < 0.001
BaKO
Naïve
Peri-tumoral vs naive
NES = 2.04
NOM p-val < 0.001
Peritumoral
Naïve
c
Oncogenic signatures (BaKO vs Naïve)
RPS14_DN.V1_UP
P53_DN.V1_UP
SNF5_DN.V1_UP
BCAT_GDS748_UP
LEF1_UP.V1_UP
MEK_UP.V1_UP
MEL18_DN.V1_DN
STK33_SKM_UP
ESC_J1_UP_EARLY.V1_DN
BMI1_DN.V1_UP
KRAS.LUNG_UP.V1_DN
CAHOY_ASTROGLIAL
ESC_V6.5_UP_EARLY.V1_DN
YAP_CONSERVED_SIGNATURE
EGFR_UP.V1_UP
KRAS.600_UP.V1_DN
KRAS.300_UP.V1_DN
ERBB2_UP.V1_UP
AKT_UP_MTOR_DN.V1_UP
LEF1_UP.V1_DN
ESC_J1_UP_LATE.V1_UP
BCAT.100_UP.V1_UP
BMI1_DN.V1_DN
P53_DN.V2_UP
ESC_J1_UP_EARLY.V1_UP
STK33_UP
MEL18_DN.V1_UP
PTEN_DN.V1_DN
BMI1_DN_MEL18_DN.V1_DN
AKT_UP.V1_UP
KRAS.LUNG.BREAST_UP.V1_DN
HOXA9_DN.V1_UP
E2F1_UP.V1_DN
CTIP_DN.V1_DN
CYCLIN_D1_KE_.V1_DN
KRAS.KIDNEY_UP.V1_DN
STK33_NOMO_UP
RB_DN.V1_UP
CRX_DN.V1_DN
IL2_UP.V1_UP
NES
NES > 1, p-val < 0.01, FDR < 0.05
d
YAP Conserved Signature
WT
BaKO
GSEA:CORDENONSI_YAP_CONSERVED_SIGNATURE
-2 0 2
e
iWAT
WT
BaKO
YAP
TAZ
Vinculin
75 kD
50 kD
125 kD
f
YAP downstream
Ctrl
Co-culture
Relative mRNA expression
Areg
Cyr61
Itgb2
Ankrd
Birc5
Ctgf
g
YAP downstream
WT
KO
Relative mRNA expression
BECN1
ANKRD
AREG
BIRC5
CTGF
CYR61
ITGB2
h
Adipogenesis
WT
KO
Relative mRNA expression
BECN1
ADIPONECTIN
AP2
CEBPα
FABP4
FASN
PPARγ
i
WT
KO
BECN1
p62
HSL
p565-HSL (Inhibition)
p563-HSL (Activation)
p660-HSL (Activation)
Actin
60 kD
60 kD
75 kD
75 kD
75 kD
75 kD
50 kD
j
FFA Concentration in CM
p=0.0145
p=0.00616
WT
KO
Concentration (μM)
#1
#6

Supplementary Figure 3: Similar characteristics observed between CAAs and BECN1-deficient adipocytes, including YAP/TAZ signaling. (a) Representative IF image of LCN2 in preadipocytes, differentiated adipocytes, and co-cultured adipocytes. 10T1/2 adipocytes were co-cultured with EO771 for 2 days. (b) GSEA plots for EMT gene set compared between naïve, peritumoral, and BaKO WAT. Normalized enrichment score (NES) and normalized (NOM) p-val are as indicated. (c) List of differentially expressed GSEA 'C6 oncogenic signature' gene sets of BaKO and naïve adipose tissue ranked by NES. Gene sets associated β-catenin and YAP are colored in blue. (d) Heatmap analysis of YAP conserved signatures between RNA-seq results of WT and BaKO iWATs. (e) Western blotting analysis of protein lysates isolated from WT and BaKO iWAT (n = 4, independent mice per group). (f) Relative mRNA expression of YAP downstream genes from adipocytes co-cultured with EO771 for 4 days (n = 3) (g) Relative mRNA expression of YAP downstream genes between WT and BECN1 KO adipocytes (n = 3). (h) Relative mRNA expression of adipogenesis-associated genes between WT and BECN1 KO adipocytes (n = 3). (i) Western blotting analysis of protein lysates isolated from WT and BaKO iWAT. (j) Free fatty acid concentration measured in media extracted from WT and BECN1 KO adipocytes (n = 4, independent cells per group).

Statistics were calculated using two-tailed unpaired students *t*-test (f, g, h, and j). Data are shown as mean $\pm$ SEM; *$p \leq 0.05$, **$p \leq 0.01$, ***$p \leq 0.001$. Western blot results are representatives of at least three independent experiments.

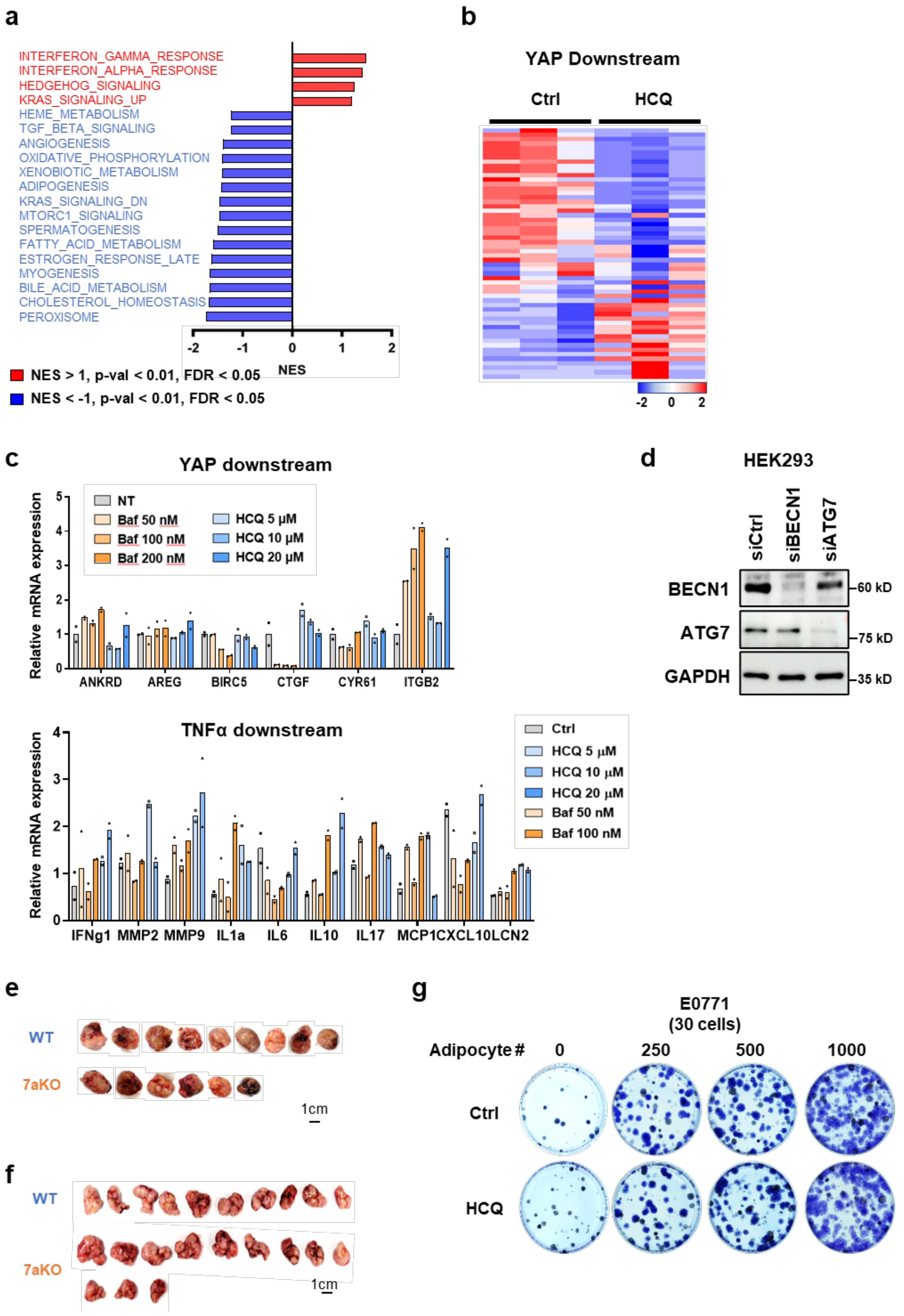

a
INTERFERON_GAMMA_RESPONSE
INTERFERON_ALPHA_RESPONSE
HEDGEHOG_SIGNALING
KRAS_SIGNALING_UP
HEME_METABOLISM
TGF_BETA_SIGNALING
ANGIOGENESIS
OXIDATIVE_PHOSPHORYLATION
XENOBIOTIC_METABOLISM
ADIPOGENESIS
KRAS_SIGNALING_DN
MTORC1_SIGNALING
SPERMATOGENESIS
FATTY_ACID_METABOLISM
ESTROGEN_RESPONSE_LATE
MYOGENESIS
BILE_ACID_METABOLISM
CHOLESTEROL_HOMEOSTASIS
PEROXISOME
NES
NES > 1, p-val < 0.01, FDR < 0.05
NES < -1, p-val < 0.01, FDR < 0.05
b
YAP Downstream
Ctrl
HCQ
c
YAP downstream
Relative mRNA expression
NT
Baf 50 nM
Baf 100 nM
Baf 200 nM
HCQ 5 μM
HCQ 10 μM
HCQ 20 μM
ANKRD
AREG
BIRC5
CTGF
CYR61
ITGB2
TNFα downstream
Ctrl
HCQ 5 μM
HCQ 10 μM
HCQ 20 μM
Baf 50 nM
Baf 100 nM
IFNg1
MMP2
MMP9
IL1a
IL6
IL10
IL17
MCP1
CXCL10
LCN2
d
HEK293
siCtrl
siBECN1
siATG7
BECN1
ATG7
GAPDH
60 kD
75 kD
35 kD
e
WT
7aKO
1cm
f
WT
7aKO
1cm
g
E0771
(30 cells)
Adipocyte #
0
250
500
1000
Ctrl
HCQ

Supplementary Figure 4: BECN1 depletion leads to adipocyte transformation. (a) GSEA of hallmark gene sets from RNA-seq results of 10T1/2 adipocytes treated with or without HCQ (20 μM, 48 hr) (n = 3, independent samples per group). (b) Heatmap analysis of YAP conserved signature gene set from RNA-seq results of 10T1/2 adipocytes treated with or without HCQ (20 μM, 48 hr) (n = 3). (c) Relative mRNA expression of YAP and TNFα downstream genes from adipocytes treated with Bafilomycin (Baf) or HCQ for 24 hrs (n = 2, independent samples per group). (d) Western blotting analysis of protein lysates isolated from HEK293 cells transfected with siBECN1 or siATG7 for Fig. 4b (n = 2, independent samples per group). (e, f) Representative image of resected tumors presented in Fig. 4c and d, respectively. (g) Colony assay of EO771 co-cultured with adipocytes treated with or without HCQ. HCQ was pretreated (20 μM, 48 hr) to the number of adipocytes as indicated.

Western blot results are representatives of at least three independent experiments.

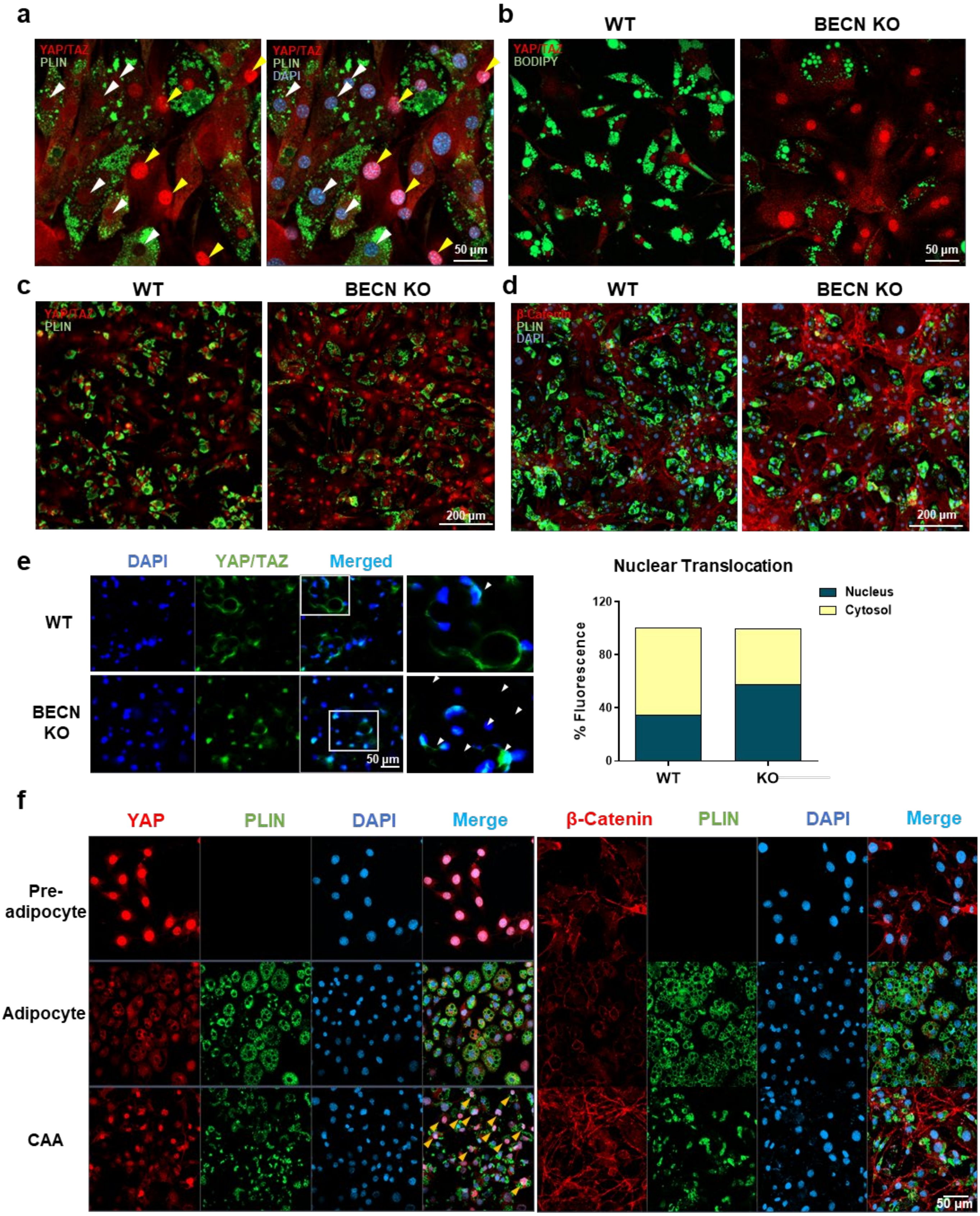
a
YAP/TAZ
PLIN
DAPI
50 µm
b
WT
BECN KO
BODIPY
c
d
β-Catenin
200 µm
e
DAPI
YAP/TAZ
Merged
BECN KO
Nuclear Translocation
Nucleus
Cytosol
% Fluorescence
120
80
40
0
KO
f
YAP
Merge
Pre-adipocyte
Adipocyte
CAA

Supplementary Figure5: Dynamic YAP/TAZ regulation modulates differentiation status of adipocytes. (a) Representative IF image of mature adipocytes stained with YAP/TAZ (red), PLIN1 (green), and DAPI (blue); white arrowheads indicate fully differentiated adipocytes (PLIN1 positive) with less nuclear YAP/TAZ; yellow arrowheads indicate undifferentiated adipocytes (PLIN negative) with nuclear YAP/TAZ. (b) Representative IF image of doxycycline-inducible *Becn1 KO* adipocytes stained with BODIPY and YAP/TAZ. (c) Representative IF image of doxycycline-inducible *Becn1 KO* adipocytes stained with YAP/TAZ and β-catenin (d) with lower magnification (Fig. 5d, e). (e) Representative IF image of fully differentiated imSVCs treated with or without 4-OHT. Adipocytes were stained with YAP/TAZ and DAPI. Nuclear translocation of YAP/TAZ is quantified using imageJ. (f) Representative IF image of 10T/2 cells as preadipocytes, differentiated adipocytes, and CAAs. Adipocytes were co-cultured with EO771 for 2 days. Yellow arrows indicate nuclear translocation of YAP in CAAs.

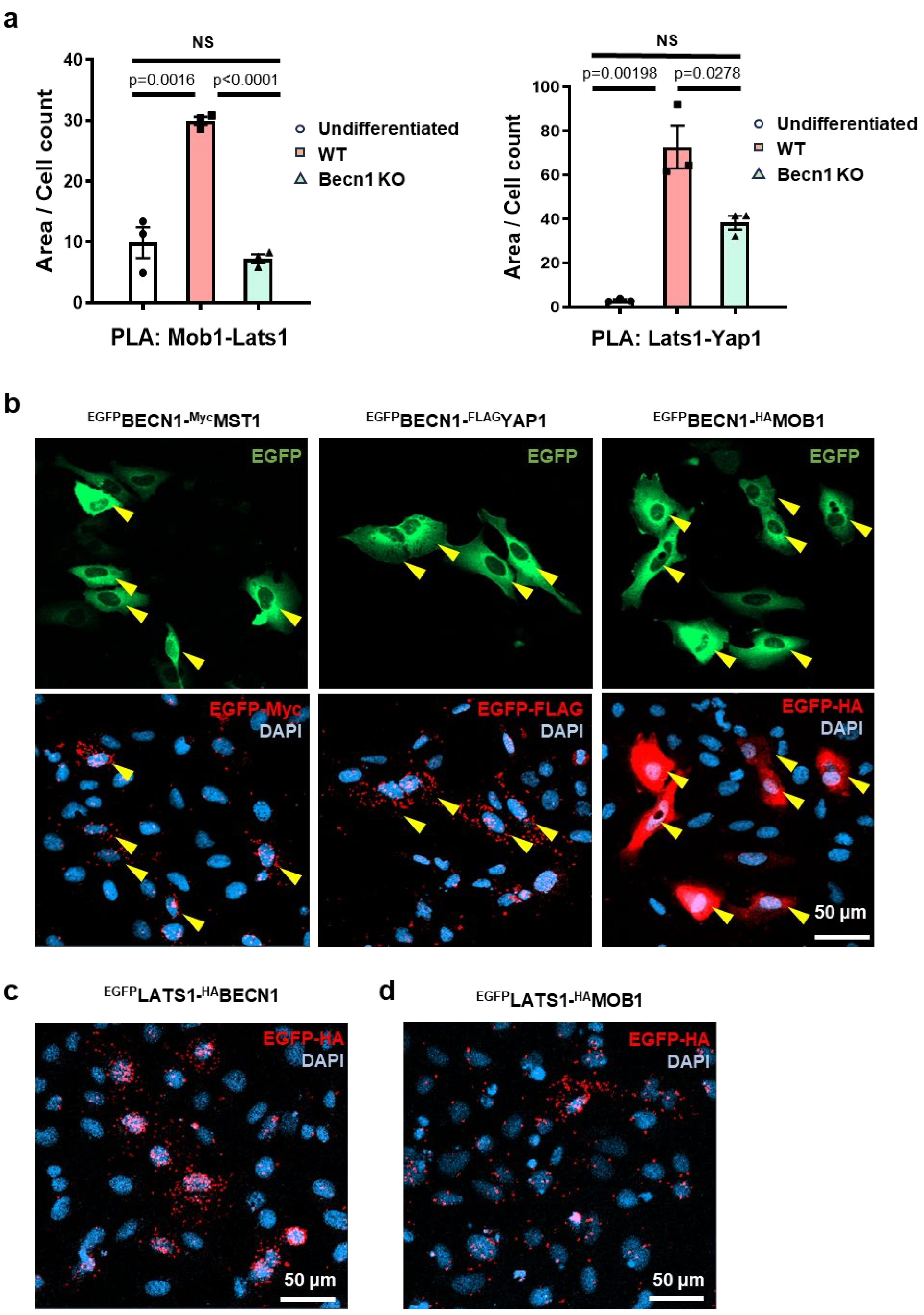
a
NS
p=0.0016
p<0.0001
Area / Cell count
Undifferentiated
WT
Becn1 KO
PLA: Mob1-Lats1
NS
p=0.00198
p=0.0278
Area / Cell count
Undifferentiated
WT
Becn1 KO
PLA: Lats1-Yap1
b
EGFPBECN1-MycMST1
EGFPBECN1-FLAGYAP1
EGFPBECN1-HAMOB1
EGFP
EGFP-Myc
EGFP-FLAG
EGFP-HA
DAPI
50 µm
c
EGFPLATS1-HABECN1
d
EGFPLATS1-HAMOB1
EGFP-HA
DAPI
50 µm

Supplementary Figure 6: BECN1 enables YAP/TAZ-mediated dedifferentiation through MOB1. (a) Quantification of PLA: MOB1-LATS1 and LATS1-YAP1 result in Fig. 5f (n = 3, independent cells per group)**.** (b) Representative IF image of PLA conducted on U2OS cells overexpressed with $BECN1^{EGFP}$ and $MST1^{MYC}$, $YAP1^{FLAG}$, and $MOB1^{HA}$ overexpressed cells are marked with yellow arrowheads. (c) Representative IF image of PLA (BECN1-LATS1 interaction) conducted on U2OS cells overexpressed with $BECN1^{HA}$ and $LATS^{EGFP}$. (d) Representative IF image of PLA (LATS1-MOB1 interaction) conducted on U2OS cells overexpressed with $LATS1^{EGFP}$ and $MOB1^{HA}$.

Statistics were calculated using two-tailed unpaired students *t*-test (a). Data are shown as mean $\pm$ SEM; *$p \leq 0.05$, **$p \leq 0.01$, ***$p \leq 0.001$.

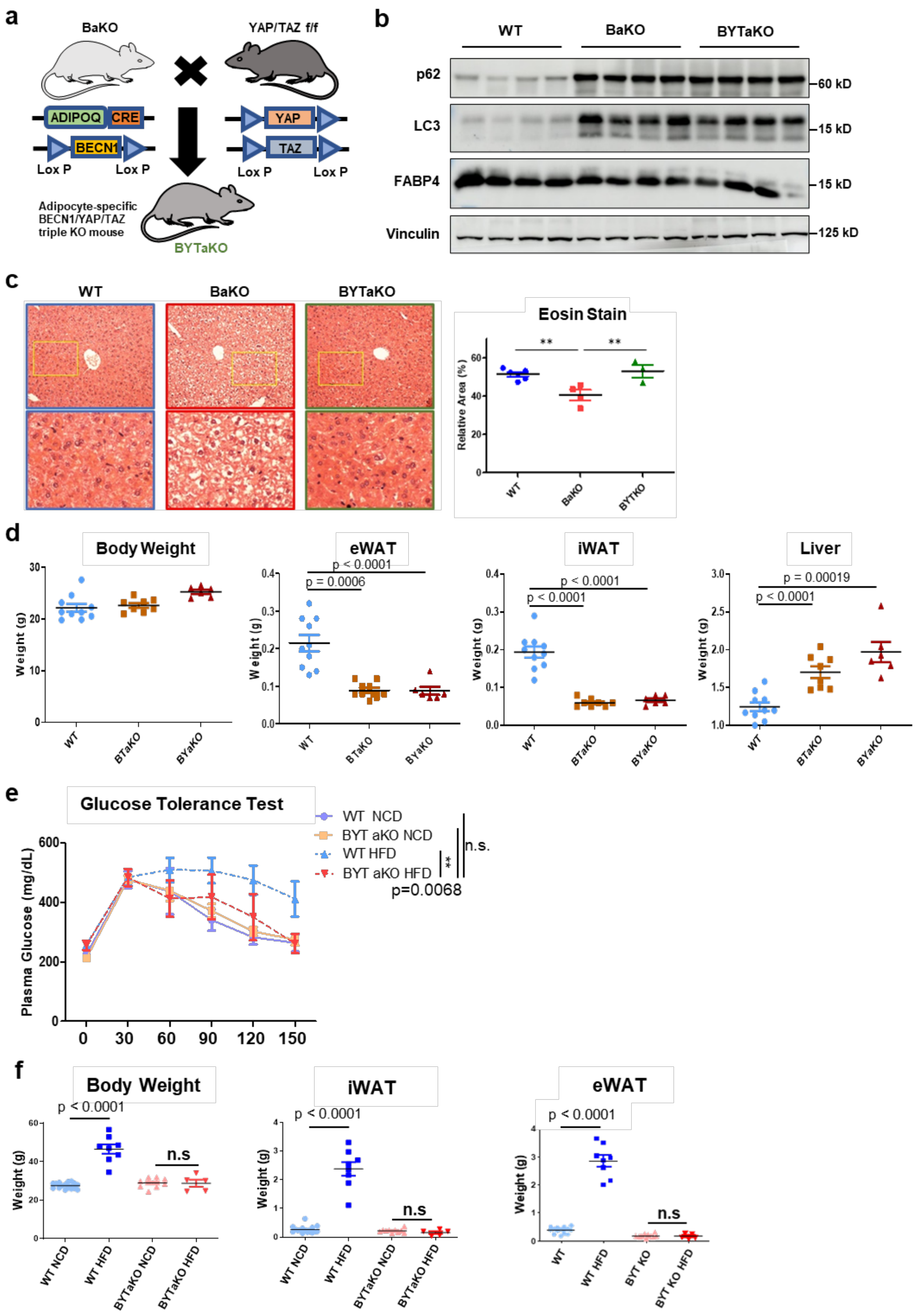
a
BaKO
YAP/TAZ f/f
ADIPOQ CRE
BECN1
YAP
TAZ
Lox P
Lox P
Lox P
Lox P
Adipocyte-specific BECN1/YAP/TAZ triple KO mouse
BYTaKO
b
WT
BaKO
BYTaKO
p62
LC3
FABP4
Vinculin
60 kD
15 kD
15 kD
125 kD
c
WT
BaKO
BYTaKO
Eosin Stain
Relative Area (%)
WT
BaKO
BYTKO
d
Body Weight
eWAT
iWAT
Liver
Weight (g)
WT
BTaKO
BYaKO
p < 0.0001
p = 0.0006
p < 0.0001
p < 0.0001
p = 0.00019
p < 0.0001
e
Glucose Tolerance Test
Plasma Glucose (mg/dL)
WT NCD
BYT aKO NCD
WT HFD
BYT aKO HFD
n.s.
**
p=0.0068
f
Body Weight
iWAT
eWAT
Weight (g)
p < 0.0001
n.s
WT NCD
WT HFD
BYTaKO NCD
BYTaKO HFD
WT
BYT KO
BYT KO HFD

Supplementary Figure 7: Additional YAP/TAZ deletion restores lipodystrophy phenotypes of BaKO. (a) Mouse breeding strategy to generate adipocyte-specific BECN1/YAP/TAZ KO (BYTaKO) mice. (b) Western blot analysis of protein isolated from WT, BaKO, and BYTaKO iWATs (8-week-old). (c) Hematoxylin and eosin staining of tissue section prepared from WT, BaKO, and BYTaKO liver (12-week-old) (WT n = 6; BaKO n = 4; BYTaKO n = 3, independent mice per group). (d) Body and organ weights measured from WT, BTaKO, and BYaKO mice (8-week-old) (WT n = 10; BaKO n = 8; BYTaKO n = 6). (e) Glucose tolerance test performed on WT and BYTaKO mice after feeding with NCD or HFD for six weeks (WT, n = 4, BYTaKO n = 6, WT HFD n = 6, BYTaKO HFD n = 5). (f) Body and organ weights measured from WT and BYTaKO mice after feeding with NCD or HFD for six weeks (WT NCD n = 16; WT HFD n = 8; BYTaKO NCD n = 10; BYTaKO HFD n = 5).

Statistics were calculated using two-tailed unpaired Student's *t*-test (c, d, and f) and ordinary two-way ANOVA (e). Data are shown as mean $\pm$ SEM; *$p \leq 0.05$, **$p \leq 0.01$, ***$p \leq 0.001$. Western blot results are representatives of at least three independent experiments.

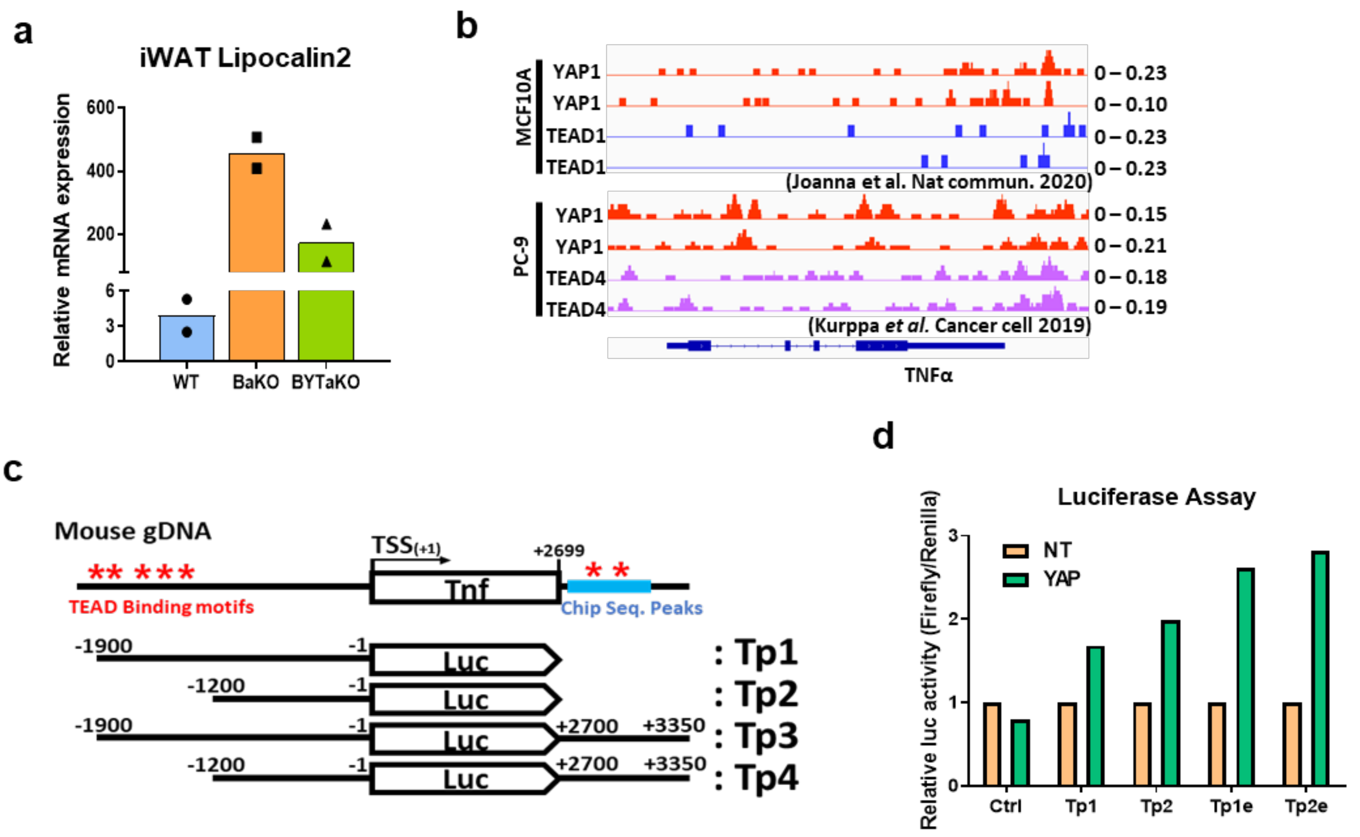

Supplementary Figure 8: YAP activity regulates LCN2 but not TNFα. (a) Relative mRNA expression of *Lcn2* in WT, BaKO, BYTaKO iWAT (n = 2, independent mice per group)**.** (b) Publicly available YAP1, TEAD1, and TEAD4 CHIP-seq reads around TNFα gene loci. CHIP-seq was conducted in MCF10A and PC-9 cells. (c) Schematic representation of luciferase reporter construct containing TNFα promoter region. Red stars indicate the TEAD binding motifs, and blue bar indicates the regions with high CHIP-seq reads. (d) Dual Luciferase assay conducted in HEK293 cells transfected with the luciferase reporter constructs (TNFα). After transfection, recombinant YAP1 protein was treated (1 μg, 48 hr).

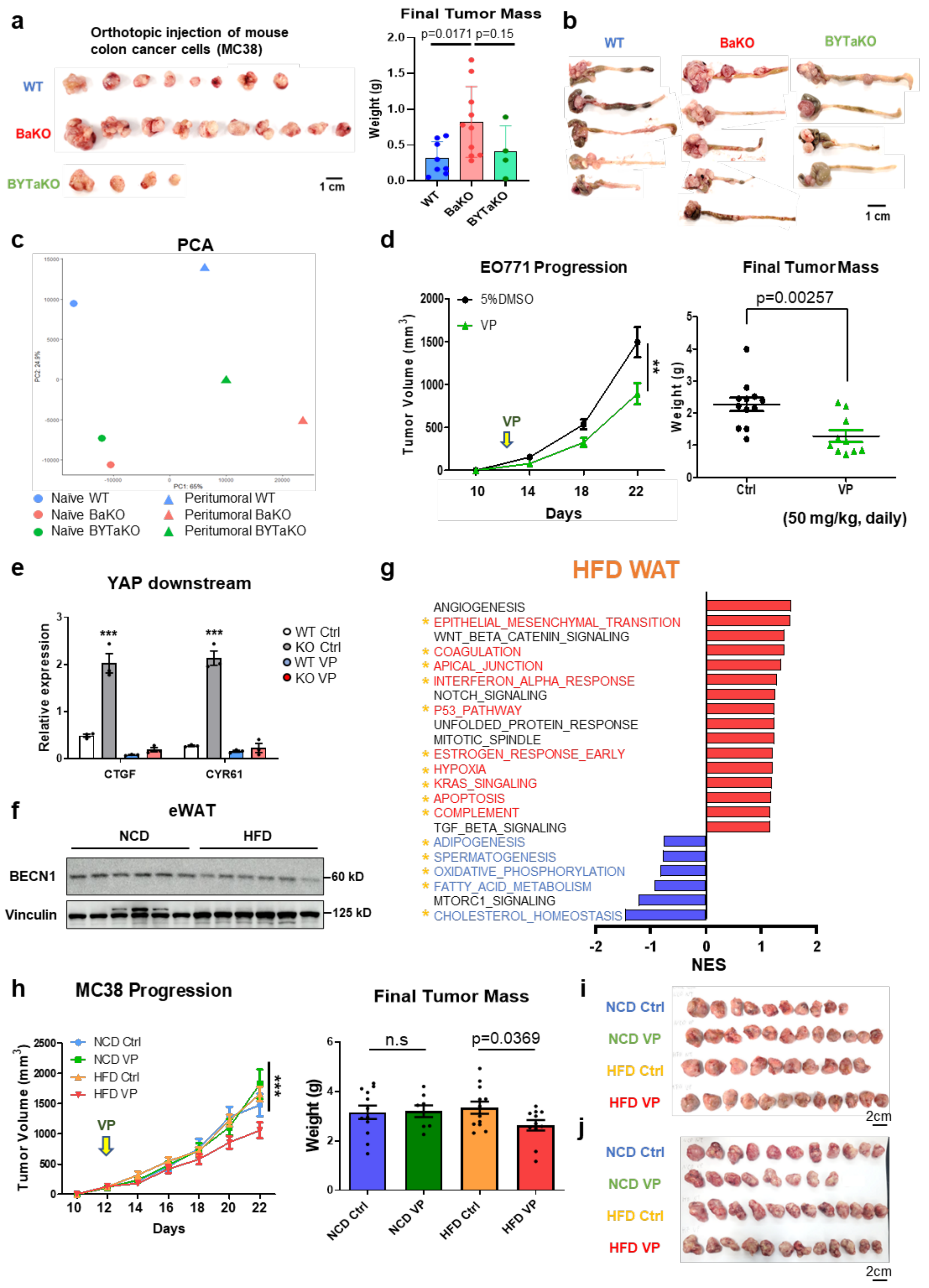

a
Orthotopic injection of mouse colon cancer cells (MC38)
WT
BaKO
BYTaKO
1 cm
Final Tumor Mass
p=0.0171 p=0.15
Weight (g)
2.0
1.5
1.0
0.5
0.0
WT
BaKO
BYTaKO
b
WT
BaKO
BYTaKO
1 cm
c
PCA
PC1: 65%
PC2: 24.9%
Naïve WT
Naïve BaKO
Naïve BYTaKO
Peritumoral WT
Peritumoral BaKO
Peritumoral BYTaKO
d
EO771 Progression
2000
1500
1000
500
0
5%DMSO
VP
Tumor Volume (mm^3)
**
10
14
18
22
Days
Final Tumor Mass
p=0.00257
Weight (g)
5
4
3
2
1
0
Ctrl
VP
(50 mg/kg, daily)
e
YAP downstream
Relative expression
3
2
1
0
***
***
CTGF
CYR61
WT Ctrl
KO Ctrl
WT VP
KO VP
f
eWAT
NCD
HFD
BECN1
Vinculin
60 kD
125 kD
g
HFD WAT
ANGIOGENESIS
* EPITHELIAL_MESENCHYMAL_TRANSITION
WNT_BETA_CATENIN_SIGNALING
* COAGULATION
* APICAL_JUNCTION
* INTERFERON_ALPHA_RESPONSE
NOTCH_SIGNALING
* P53_PATHWAY
UNFOLDED_PROTEIN_RESPONSE
MITOTIC_SPINDLE
* ESTROGEN_RESPONSE_EARLY
* HYPOXIA
* KRAS_SINGALING
* APOPTOSIS
* COMPLEMENT
TGF_BETA_SIGNALING
* ADIPOGENESIS
* SPERMATOGENESIS
* OXIDATIVE_PHOSPHORYLATION
* FATTY_ACID_METABOLISM
MTORC1_SIGNALING
* CHOLESTEROL_HOMEOSTASIS
-2
-1
0
1
2
NES
h
MC38 Progression
NCD Ctrl
NCD VP
HFD Ctrl
HFD VP
2500
2000
1500
1000
500
0
Tumor Volume (mm^3)
VP
***
10
12
14
16
18
20
22
Days
Final Tumor Mass
n.s
p=0.0369
Weight (g)
6
4
2
0
NCD Ctrl
NCD VP
HFD Ctrl
HFD VP
i
NCD Ctrl
NCD VP
HFD Ctrl
HFD VP
2cm
j
NCD Ctrl
NCD VP
HFD Ctrl
HFD VP
2cm

Supplementary Figure 9: Deactivation of adipocyte YAP/TAZ represses malignant TME formation. (a) Colon orthotopic injection of MC38 into mouse cecum. Mice were sacrificed after 4 weeks post-injection. Final tumor mass measured after resection of each tumor (WT n = 8, BaKO n = 10, BYTaKO n = 4). (b) Image of resected colons harboring tumors. (c) Principal component analysis performed on RNA-seq results of WT, BaKO, and BYTaKO WATs. Peritumoral adipose tissues were extracted two weeks after MC-38 injection (n = 2, independent sequencing results per group). (d) Tumor volumes and weights after mammary fat pad injection of EO771 into WT mice. VP treatment (50 mg/kg, injected everyday) was initiated when tumors reached a volume of 150 mm^3, and the mice were sacrificed on day 22 following the injection (Ctrl n = 12; VP n = 10). (e) Relative mRNA expression of YAP downstream in WT and *Becn1* KO imSVCs treated with or without VP (20 nM) for 24 h (n = 3, independent samples per group). (f) Western blot analysis of protein lysates isolated from eWAT of NCD and HFD fed mice. (g) GSEA result of HFD-fed mouse and NCD-fed mouse iWATs ranked by NES. Gene sets that also appear to be differentially expressed in BaKO iWAT are indicated with yellow stars. (h) Tumor volumes and weights after subcutaneous injection of MC-38 into NCD and HFD-fed mice. VP treatment (30 mg/kg) was initiated when tumors reached a volume of 300 mm^3, and the mice were sacrificed on day 22 following the injection (NCD Ctrl n = 12; NCD VP n = 9; HFD Ctrl n = 12; HFD VP n = 11). (i, j) Image of tumors resected from Fig. 7g (i) and Supplementary Fig. 9f (j).

Statistics were calculated using two-tailed unpaired Student's *t*-test (a, d, h), ordinary two-way ANOVA (d and h), and one-way ANOVA (e). Data are shown as mean $\pm$ SEM; *$p \leq 0.05$, **$p \leq 0.01$, ***$p \leq 0.001$. Western blot results are representatives of at least three independent experiments.

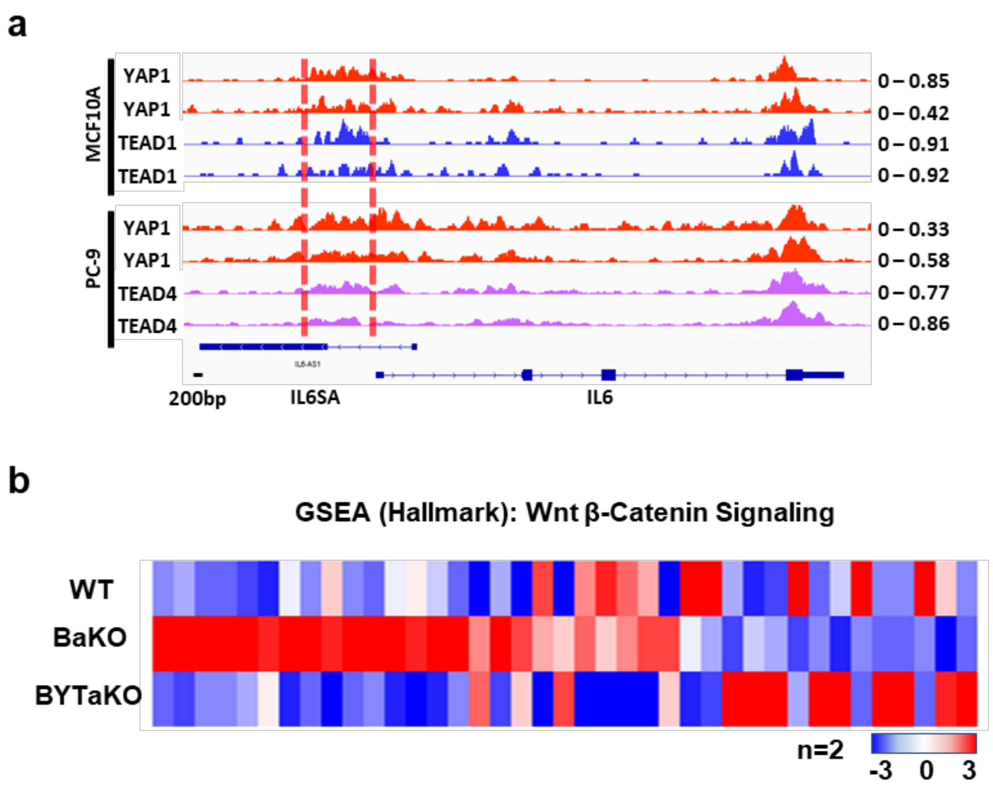

Supplementary Figure 10: IL6 - Wnt/β-catenin signaling in BaKO and BYTaKO iWATs. (a) Publicly available YAP1, TEAD1, and TEAD4 CHIP-seq reads around IL6 gene loci. CHIP-seq was conducted in MCF10A and PC-9 cells. (b) Heatmap analysis of Wnt/β-catenin signaling gene set from RNA-seq results of WT, BaKO, and BYTaKO iWATs (n = 2, independent samples per group).

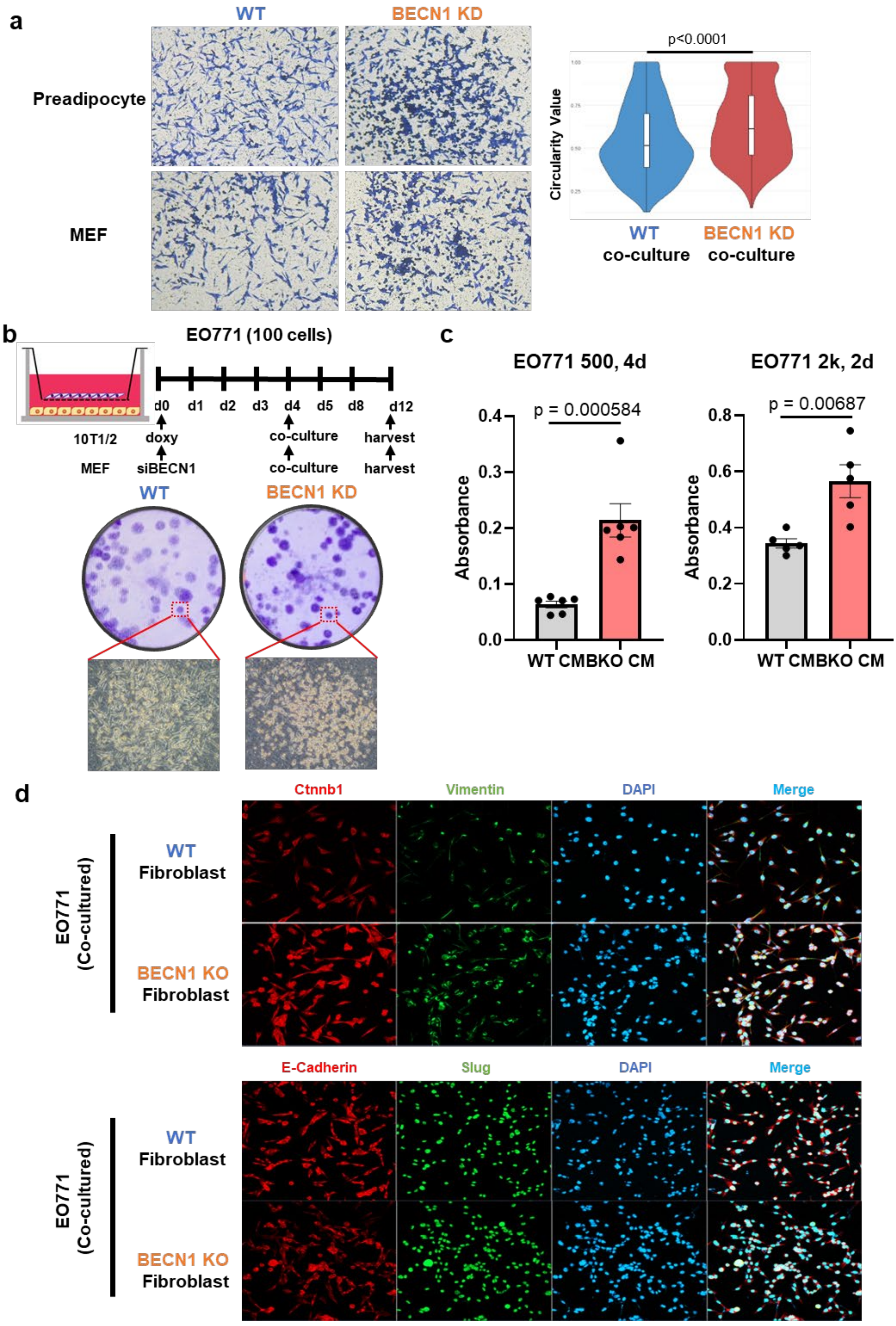
a
WT
BECN1 KD
Preadipocyte
MEF
p<0.0001
Circularity Value
WT co-culture
BECN1 KD co-culture
b
EO771 (100 cells)
d0 d1 d2 d3 d4 d5 d8 d12
10T1/2
doxy
co-culture
harvest
MEF
siBECN1
co-culture
harvest
WT
BECN1 KD
c
EO771 500, 4d
p = 0.000584
Absorbance
WT CM
BKO CM
EO771 2k, 2d
p = 0.00687
Absorbance
WT CM
BKO CM
d
Ctnnb1
Vimentin
DAPI
Merge
EO771 (Co-cultured)
WT Fibroblast
BECN1 KO Fibroblast
E-Cadherin
Slug
DAPI
Merge
EO771 (Co-cultured)
WT Fibroblast
BECN1 KO Fibroblast

Supplementary Figure 11: Depletion of fibroblast-BECN1 induces tumor growth and EMT. (a) Crystal violet staining of EO771 cells co-cultured with preadipocytes or MEFs. BECN1 was depleted from both preadipocytes and MEF prior to the co-culture. Circularity of each cell was measured using imageJ to represent cellular morphologies (WT n = 1157, BECN1 KD n = 975, individual cells per group). (b) Schematic timeline of BECN1 depletion in preadipocytes and MEFs. DIC image of EO771 co-cultured with WT or BECN1-deficient preadipocytes. (c) MTT assay results of EO771 grown in preadipocyte-derived CM. BECN1 was depleted by doxycycline treatment. The CM was collected every 4 days of incubation (n = 6, independent cells per group). (d) Representative IF image of EO771 co-cultured with WT or BECN1-deficient fibroblasts stained with EMT markers. EO771 cells were co-cultured for 2 days and stained with β-catenin, Vimentin, E-cadherin, and Slug.

Statistics were calculated using two-tailed unpaired students *t*-test (a and c). Data are shown as mean $\pm$ SEM; *$p \leq 0.05$, **$p \leq 0.01$, ***$p \leq 0.001$.

Supplementary table 1: Sequences of genotyping primers used in this study.

	Forward	Reverse
Actin	GTAGGTGGAAATTCTAGCATCATCC	CTAGGCCACAGAATTGAAAGATCT
Adipoq-Cre	ACCTGAAGATGTTCGCGATTATCT	ACCGTCAGTACGTGAGATATCTT
BECN1f/f	GCTTAGGTCTGACTGGGTCG	ACCCAGGCAAGTGTGTTTTC
YAP1f/f	ACATGTAGGTCTGCATGCCAGAGGAGG	AGGCTGAGACAGGAGGATCTCTGTGAG
TAZf/f	TTGTGACAAAGAACCTGGGGCTATC	TGAGGAACTCTTCCATTGCCAGG
ATG7f/f	TGGCTGCTATTTCTGCAATG	TTCTGGATGCTGCAAAACAG
MMTV-PyMT	TGGCGGCCTCTTGACTATGAT	ATCTGTGCTTATGCCTATGCCCGC

Supplementary table 2: shRNA sequences used to knockdown BECN1, YAP, and TAZ.

sh_BECN1_1	AAAAGGTTTGGAAAGATGCTTTATTGGATCCAATAAAGCATCTTTCCAAACC
sh_BECN1_2	AAAAGGATTTGCTATGGTCTATTTTGGATCCAAAATAGACCATAGCAAATCC
sh_BECN1_3	AAAAGGTGATGAATTCTCATCTATTGGATCCAATAGATGAGAATTCATCACC
sh_BECN1_4	AAAAGGAAAGCTGTTTCCTTATATTGGATCCAATATAAGGAAACAGCTTTCC
sh_BECN1_5	AAAAGGTAAAAGGTGCTGTCAAATTGGATCCAATTTGACAGCACCTTTTACC
sh_YAP_1	AAAAGGTTTGGAAAGATGCTTTATTGGATCCAATAAAGCATCTTTCCAAACC
sh_YAP_2	AAAAGGATTTGCTATGGTCTATTTTGGATCCAAAATAGACCATAGCAAATCC
sh_YAP_3	AAAAGGTGATGAATTCTCATCTATTGGATCCAATAGATGAGAATTCATCACC
sh_YAP_4	AAAAGGAAAGCTGTTTCCTTATATTGGATCCAATATAAGGAAACAGCTTTCC
sh_YAP_5	AAAAGGTAAAAGGTGCTGTCAAATTGGATCCAATTTGACAGCACCTTTTACC
sh_TAZ_1	AAAAGCATTTCTGTGGCAGATAATTGGATCCAATTATCTGCCACAGAAATGC
sh_TAZ_2	AAAAGCCTGATGAACCATGAATTTTGGATCCAAAATTCATGGTTCATCAGGC
sh_TAZ_3	AAAAGCCAGGTGATGTGAAATTATTGGATCCAATAATTTCACATCACCTGGC
sh_TAZ_4	AAAAGCCCTTGACTGTTTACTAATTGGATCCAATTAGTAAACAGTCAAGGGC
sh_TAZ_5	AAAAGCACCTTGTGAAAGTGTAATTGGATCCAATTACACTTTCACAAGGTGC

Supplementary table 3: siRNA sequences used to knockdown LCN2R.

	Sense	Anti-sense
si_LCN2R1	GCAGAGGGCU AGAAAGAUA	UAUCUUUCUA GCCCUCUGC
si_LCN2R2	GCAGAGGGCU AGAAAGAUA	UAUCUUUCUA GCCCUCUGC
si_LCN2R3	CGCUAUAACC AUUAGGUCU	AGACCUAAUG GUUAUAGCG
si_LCN2R4	GCUAUAACCA UUAGGUCUG	CAGACCUAAU GGUUAUAGC

Supplementary table 4: Sequences of mouse RT-qPCR primers used in this study.

	Forward	Reverse
BECN1	CAGCCTCTGAAACTGGACACGA	CTCTCCTGAGTTAGCCTCTTCC
Adiponectin	GCAGAGATGGCACTCCTGGA	CCCTTCAGCTCCTGTCATTCC
AP2/FABP4	TGAAATCACCGCAGACGACAGG	GCTTGTCACCATCTCGTTTTCTC
CEBPalpha	GGGTGAGTTCATGGAGAATGG	CAGTTTGGCAAGAATCAGAGCA
FASN	CACAGTGCTCAAAGGACATGCC	CACCAGGTGTAGTGCCTTCCTC
PPARγ	TGCACTGCCTATGAGCACTT	GAATGCGAGTGGTCTTCCAT
CTGF	GTGCCAGAACGCACACTG	CCCCGGTTACACTCCAAA
CYR61	AGAGGCTTCCTGTCTTTGGC	CCAAGACGTGGTCTGAACGA
AREG	TCATGGCGAATGCAGATACA	GCTACTACTGCAATCTTGGA
ITGB2	GCTTTGGGTCGTTTGTGGAC	TGCCGACCTCTGTCTGAAAC
ANKRD	GGAACAACGGAAAAGCGAGAA	GAAACCTCGGCACATCCACA
BIRC5	TGCAAAGGAGACCAACAACA	GGCATGTCACTCAGGTCCAA
TNFalpha	CCCCAAAGGGATGAGAAGTT	CACTTGGTGGTTTGCTACGA
IFNgamma	GCTCTTCCTCATGGCTGTTT	GTCACCATCCTTTTGCCAGT
MMP2	AGATCTTCTTCTTCAAGGACCGGTT	GGCTCCTCAGTGGCTTGGGGTA
MMP9	TGAATCAGCTGGCTTTTGTG	ACCTTCCAGTAGGGGCAACT
IL6	TGAGAAAAGAGTTGTGCAATGG	GGTACTCCAGAAGACCAGAGG
IL-17alpha	CCAGGGAGAGCTTCATCTGT	AGGAAGTCCTTGGCCTCAGT
MCP1/CCL2	GCTACAAGAGGATCACCAGCAG	GTCTGGACCCATTCCTTCTTGG
SerpinE1	CCTCTTCCACAAGTCTGATGGC	GCAGTTCCACAACGTCATACTCG
Resistin	TCACTTTTCACCTCTGTGGATATGAT	TGCCCCAGGTGGTGTAAA
Lipocalin2	TGCCACTCCATCTTTCCTGTT	GGGAGTGCTGGCCAAATAAG
CRP	GATTCCTGAGGCTCCAACACAC	ACAGTGTAGCCCTTGTGCAGAC
IGF1	GTGGATGCTCTTCAGTTCGTGTG	TCCAGTCTCCTCAGATCACAGC
Pentraxin3	CGAAATAGACAATGGACTTCATCC	CATCTGCGAGTTCTCCAGCATG
Endocan	CTGGAGAAACCTGCTACCGTAC	CATTCCATCCCGAAGGTGCCAT
Adiposin	CATGCTCGGCCCTACATGG	CACAGAGTCGTCATCCGTCAC
Apelin	CACTGATGTTGCCTCCAGATGGA	ACGCCATTAGACGAACTTGGTGG
Chimerin	CTTCTCCCGTTTGGTTTGATTG	TACAGGTGGCTCTGGAGGAGTTC
Leptin	TGCTGCAGATAGCCAATGAC	GAGTAGAGTGAGGCTTCCAGG
OncostatinM	GCACGGGCCAGAGTACCAGGAC	CTGGTGTTGTAGTGGACCGTGAG
RAGE	GCCACTGGAATTGTCGATGAGG	GCTGTGAGTTCAGAGGCAGGAT
YAP	ACATGTAGGTCTGCATGCCAGAGG	AGGCTGAGACAGGAGGATCTCTGT
TAZ	TCCTCTGTGTGTGTGCTGAG	CTGAAACTACAATAAACTCTCCC
IL8	ATGGCTGCTCAAGGCTGGTC	AGGCTTTTCATGCTCAACACTAT
IL10	GACCAGCTGGACAACATACTGCTAA	GATAAGGCTTGGCAACCCAAGTAA
