# Supplementary material for: Dysfunctional adipocytes promote tumor progression through YAP/TAZ-dependent cancer-associated adipocyte transformation
Source: Nat Commun. 2024 May 14;15:4052. doi: 10.1038/s41467-024-48179-3 (PMC11094189; doi:10.1038/s41467-024-48179-3)
Supplement: Supplementary file 2 — Reporting Summary [file 41467_2024_48179_MOESM2_ESM.pdf]

nature portfolio

Corresponding author(s): Han-Woong Lee

Last updated by author(s): Jul 3, 2023

Reporting Summary

Nature Portfolio wishes to improve the reproducibility of the work that we publish. This form provides structure for consistency and transparency in reporting. For further information on Nature Portfolio policies, see our Editorial Policies and the Editorial Policy Checklist.

Statistics

For all statistical analyses, confirm that the following items are present in the figure legend, table legend, main text, or Methods section.

n/a	Confirmed	
☐	☒	The exact sample size (*n*) for each experimental group/condition, given as a discrete number and unit of measurement
☐	☒	A statement on whether measurements were taken from distinct samples or whether the same sample was measured repeatedly
☐	☒	The statistical test(s) used AND whether they are one- or two-sided<br>*Only common tests should be described solely by name; describe more complex techniques in the Methods section.*
☐	☒	A description of all covariates tested
☐	☒	A description of any assumptions or corrections, such as tests of normality and adjustment for multiple comparisons
☐	☒	A full description of the statistical parameters including central tendency (e.g. means) or other basic estimates (e.g. regression coefficient) AND variation (e.g. standard deviation) or associated estimates of uncertainty (e.g. confidence intervals)
☐	☒	For null hypothesis testing, the test statistic (e.g. *F*, *t*, *r*) with confidence intervals, effect sizes, degrees of freedom and *P* value noted<br>*Give P values as exact values whenever suitable.*
☐	☒	For Bayesian analysis, information on the choice of priors and Markov chain Monte Carlo settings
☐	☒	For hierarchical and complex designs, identification of the appropriate level for tests and full reporting of outcomes
☒	☐	Estimates of effect sizes (e.g. Cohen's *d*, Pearson's *r*), indicating how they were calculated

Our web collection on statistics for biologists contains articles on many of the points above.

Software and code

Policy information about availability of computer code

Data collection	Real-time quantitative PCR data was collected using Biorad CFX Manager (version 3.1). Western blot data was collected using Amersham ImageQuant 800 (version 1.2.1). Immunofluorescence data was collected using ZEISS Zen microscopy software (version 3.7). MTT assay data was collected using GloMax Discover (version 3.1.0). Gene set enrichment assay data was collected using GSEA (version 4.2.3). RNA sequencing data was collected by BCL2FASTQ (version unknown).
Data analysis	Quantification of cancer cell line colony sizes and mouse adipokine array data was performed using ImageJ (version 1.52a). Graphs of tumor size, tumor mass, survival, mRNA expression, and cytokine concentration were created using GraphPad Prism (version 5.01). Heatmaps of various gene sets were generated using R software (version 4.3.1). RNA sequencing data was analyzed by RSEM/STAR (Version unknown).

For manuscripts utilizing custom algorithms or software that are central to the research but not yet described in published literature, software must be made available to editors and reviewers. We strongly encourage code deposition in a community repository (e.g. GitHub). See the Nature Portfolio guidelines for submitting code & software for further information.

Data

Policy information about availability of data

All manuscripts must include a data availability statement. This statement should provide the following information, where applicable:

- Accession codes, unique identifiers, or web links for publicly available datasets
- A description of any restrictions on data availability
- For clinical datasets or third party data, please ensure that the statement adheres to our policy

All data are available in the main text, supplementary materials or relevant repositories. RNA-seq data are uploaded in public database 'Genomic Sequence Archive (GSA)' with accession code CRA011844. Chip-seq data used in this study are available in public database 'ChIP-Atlas' with accession code SRX10119365, SRX10119369, SRX10119373, SRX10119371, SRX5887618, SRX5887617, SRX5887621, and SRX5887620. Source data are provided with this paper. Plasmids, cell lines, and other materials in this study can be shared upon request. For further information and requests for resources and reagents, please contact Han-Woong Lee (hwl@yonsei.ac.kr).

Research involving human participants, their data, or biological material

Policy information about studies with human participants or human data. See also policy information about sex, gender (identity/presentation), and sexual orientation and race, ethnicity and racism.

Reporting on sex and gender	No human patients were involved in the study.
Reporting on race, ethnicity, or other socially relevant groupings	No human patients were involved in the study.
Population characteristics	No human patients were involved in the study.
Recruitment	No human patients were involved in the study.
Ethics oversight	No human patients were involved in the study.

Note that full information on the approval of the study protocol must also be provided in the manuscript.

Field-specific reporting

Please select the one below that is the best fit for your research. If you are not sure, read the appropriate sections before making your selection.

[x] Life sciences [ ] Behavioural & social sciences [ ] Ecological, evolutionary & environmental sciences

For a reference copy of the document with all sections, see nature.com/documents/nr-reporting-summary-flat.pdf

Life sciences study design

All studies must disclose on these points even when the disclosure is negative.

Sample size	No statistical methods were used to predetermine sample size. Sample sizes were estimated based on experiences on the similar experiments performed by us and other published studies.
Data exclusions	No data was excluded.
Replication	The growth of tumors in syngeneic (EO771, MC-38, 4T1, TC-1, B16F10, LL2), and genetic (MMTV-PyMT) mouse models was validated by experiments with at least 2 separate cohorts or 2 different injection sites (orthotropic and subcutaneous). Studies using adipocytes were cross-validated in various adipocyte cell lines such as SVF-derived imSVC, 10T1/2, and 3T3L1 all with at least two separate experiments each. Real-time qPCR was performed on two separate samples with triplicate copies for each sample and all attempts at replication were successful. All the data from the experiments involving antibodies, such as western blotting, immunocytochemistry, and immunohistochemistry, were all successfully replicated in at least 2 separate experiments with different samples except western blot data of LCN2 in Fig. 2h. However, the LCN2 level was validated by other quantitative data, such as RT-qPCR and immunocytochemistry, that have successful replication. The colony size data of co-cultivated cancer cells has not been replicated, but each data includes various factors such as adipocyte numbers. All attempts at replication of luciferase data were successful. All attempts at replication of Elisa data were successful.
Randomization	Every genetically engineered mouse model that was used in the study was periodically out-bred with WT mice. The cohort of mice was allocated based on their age at the moment of the experiment. The in vivo experiments were conducted on mice with similar ages but different mothers. Every cancer cell injection or drug treatment was distributed equally and simultaneously regardless of their genotypes. During the in vitro experiment using cell lines, drug treatment, co-culture, and genetic alteration were conducted equally and simultaneously.
Blinding	Mouse phenotypes (tumor size, organ weight, insulin sensitivity, etc) were measured without any notation of the mouse's genotypes. Primary tumor size and metastatic nodules were measured by colleagues who were not involved in this research. Most in vitro experiments were not performed in blind but replication and randomization of factors were applied.

Reporting for specific materials, systems and methods

We require information from authors about some types of materials, experimental systems and methods used in many studies. Here, indicate whether each material, system or method listed is relevant to your study. If you are not sure if a list item applies to your research, read the appropriate section before selecting a response.

Materials & experimental systems

n/a	Involved in the study
☐	☒ Antibodies
☐	☒ Eukaryotic cell lines
☒	☐ Palaeontology and archaeology
☐	☒ Animals and other organisms
☒	☐ Clinical data
☒	☐ Dual use research of concern
☒	☐ Plants

Methods

n/a	Involved in the study
☒	☐ ChIP-seq
☒	☐ Flow cytometry
☒	☐ MRI-based neuroimaging

Antibodies

Antibodies used

Western blotting was performed with the following antibodies: anti-BECN1 (#3738; Cell Signaling Technology & sc-48341; Santa Cruz Biotechnology), anti-FASN (#3180; Cell Signaling Technology), anti-PPARγ (#2435; Cell Signaling Technology), anti-PLIN1 (#9349; Cell Signaling Technology), anti-actin (sc-47778; Santa Cruz Biotechnology), anti-GAPDH (sc-32233; Santa Cruz Biotechnology), anti-LC3 (L8918; Sigma-Aldrich), anti-p62 (H00008878-M01; Abnova), anti-tubulin (sc-48341; Santa Cruz Biotechnology), anti-β-catenin (sc-7963; Santa Cruz Biotechnology), anti-YAP/TAZ (sc-101199; Santa Cruz), anti-YAP (#14074; Cell Signaling Technology), anti-TAZ (#4883; Cell Signaling Technology), anti-phospho-YAP (#13008; Cell Signaling Technology), anti-LCN2 (AF1857; R&D Systems), anti-HSL (#4107; Cell Signaling Technology), anti-phospho-HSL (#4137; #4139, #45804; Cell Signaling Technology), anti-MOB1A (sc-393212; Santa Cruz Biotechnology), anti-phospho-MOB1 (#8699; Cell Signaling Technology), anti-MST1 (#3682; Cell Signaling Technology), Goat anti-Mouse IgG(H+L)-HRP (SA001; GenDEPOT), Goat anti-Rabbit IgG(H+L)-HRP (SA002; GenDEPOT), and Rabbit anti-Goat IgG(H+L)-HRP (SA007; GenDEPOT).

Validation

Validation statements from Cell Signaling Technology
"To ensure our antibodies will work in your experiment, we adhere to the Hallmarks of Antibody Validation™, six complementary strategies that can be used to determine the functionality, specificity, and sensitivity of an antibody in any given assay."
https://www.cellsignal.com/about-us/cst-antibody-validation-principles

Links to the antibody profile, relevant citations, and validation of primary antibodies from "Cell signaling technology" are attached below.

anti-BECN1 (#3738; Cell Signaling Technology)
https://www.cellsignal.com/products/primary-antibodies/beclin-1-antibody/3738

anti-FASN (#3180; Cell Signaling Technology)
https://www.cellsignal.com/products/primary-antibodies/fatty-acid-synthase-c20g5-rabbit-mab/3180

anti-PPARγ (#2435; Cell Signaling Technology)
https://www.cellsignal.com/products/primary-antibodies/pparg-c26h12-rabbit-mab/2435

anti-PLIN1 (#9349; Cell Signaling Technology)
https://www.cellsignal.com/products/primary-antibodies/perilipin-1-d1d8-xp-rabbit-mab/9349

anti-YAP (#14074; Cell Signaling Technology)
https://www.cellsignal.com/products/primary-antibodies/yap-d8h1x-xp-rabbit-mab/14074

anti-TAZ (#4883; Cell Signaling Technology)
https://www.cellsignal.com/products/primary-antibodies/taz-v386-antibody/4883

anti-phospho-YAP (#13008; Cell Signaling Technology)
https://www.cellsignal.com/products/primary-antibodies/phospho-yap-ser127-d9w2i-rabbit-mab/13008

anti-HSL (#4107; Cell Signaling Technology)
https://www.cellsignal.com/products/primary-antibodies/hsl-antibody/4107

anti-phospho-HSL (#4137, #4139, #45804; Cell Signaling Technology)
https://www.cellsignal.com/products/primary-antibodies/phospho-hsl-ser565-antibody/4137
https://www.cellsignal.com/products/primary-antibodies/phospho-hsl-ser563-antibody/4139
https://www.cellsignal.com/products/primary-antibodies/phospho-hsl-ser660-antibody/45804

anti-phospho-MOB1 (#8699, Cell signaling Technology)
https://www.cellsignal.com/products/primary-antibodies/phospho-mob1-thr35-d2f10-rabbit-mab/8699

anti-MST1 (#3682, Cell signaling Technology)
https://www.cellsignal.com/products/primary-antibodies/mst1-antibody/3682

Validation statements from Sigma Aldrich
"We therefore subsequently test in as many additional immunodetection applications as practical in samples chosen to be relevant to the intended use of the product. These include immunohistochemistry, immunocytochemistry (ICC), Western blot, ELISA, immunoprecipitation, and more."

https://www.sigmaaldrich.com/KR/ko/technical-documents/technical-article/protein-biology/immunohistochemistry/antibody-enhanced-validation

Links to the antibody profile, relevant citations, and validation of primary antibodies from "Sigma Aldrich" are attached below.
anti-LC3 (L8918; Sigma-Aldrich)
https://www.sigmaaldrich.com/KR/ko/product/sigma/l8918?utm_source=google&utm_medium=cpc&utm_campaign=13065547384&utm_content=130760041108&gclid=CjwKCAiAtt2tBhBDEiwALZuhALXKJKaTzaFJpOH74Jso3DD6isv4WizlaGwZLYBmXizGOGwZTaRZZBoCsngQAvD_BwE

Validation statements from R&D Systems
"Our antibodies continue to get validated through peer-review and are consistently published in high-impact scientific journals."

https://www.rndsystems.com/quality/antibodies-built-for-reproducibility

Links to the antibody profile, relevant citations, and validation of primary antibodies from "R&D Systems" are attached below.

anti-LCN2 (AF1857; R&D Systems)
https://www.rndsystems.com/products/mouse-lipocalin-2-ngal-antibody_af1857

Validation of antibodies based on the data provided in the manuscript.
anti-p62 (H00008878-M01; Abnova): Accumulation of P62 upon autophagy inhibitions (hydroxychloroquine and bafilomycin) was confirmed in a Figure 4 in the manuscript.

anti-BECN1 (sc-48341; Santa Cruz Biotechnology): The depletion of Becn1 by siBECN1 is presented in Figure 4 of the manuscript. The depletion of Becn1 by doxycycline-inducible shBECN1 is presented in Figure 5 of the manuscript.

Relevant citations for other antibodies
anti-actin (sc-47778; Santa Cruz Biotechnology): Lee, S., Lee, H., Baek, G. et al. Precision mitochondrial DNA editing with high-fidelity DddA-derived base editors. Nat Biotechnol 41, 378–386 (2023). https://doi.org/10.1038/s41587-022-01486-w

anti-GAPDH (sc-32233; Santa Cruz Biotechnology): Wang, L., Wang, G., Mao, W. et al. Bioinspired engineering of fusogen and targeting moiety equipped nanovesicles. Nat Commun 14, 3366 (2023). https://doi.org/10.1038/s41467-023-39181-2

anti-tubulin (sc-48341; Santa Cruz Biotechnology): Seo, J., Seong, D., Nam, Y.W. et al. Beclin 1 functions as a negative modulator of MLKL oligomerisation by integrating into the necrosome complex. Cell Death Differ 27, 3065–3081 (2020). https://doi.org/10.1038/s41418-020-0561-9

anti-β-catenin (sc-7963; Santa Cruz Biotechnology): Riascos-Bernal, D., Chinnasamy, P., Cao, L. et al. β-Catenin C-terminal signals suppress p53 and are essential for artery formation. Nat Commun 7, 12389 (2016). https://doi.org/10.1038/ncomms12389

anti-YAP/TAZ (sc-101199; Santa Cruz): Bera, K., Kiepas, A., Godet, I. et al. Extracellular fluid viscosity enhances cell migration and cancer dissemination. Nature 611, 365–373 (2022). https://doi.org/10.1038/s41586-022-05394-6

anti-MOB1A (sc-393212; santa Cruz): Praskova M, Xia F, Avruch J. MOBKL1A/MOBKL1B phosphorylation by MST1 and MST2 inhibits cell proliferation. Curr Biol. 2008 Mar 11;18(5):311-21. doi: 10.1016/j.cub.2008.02.006.

Eukaryotic cell lines

Policy information about cell lines and Sex and Gender in Research

Cell line source(s)

The 3T3L1 (#CL-173), 10T1/2 (#CCL-226), EO771 (#CRL-3461), 4T1 (#CRL-2539), B16F10 (#CRL-6475), LL/2 (#CRL-1642), TC-1 (#CRL-2493), U2OS (#HTB-96) and HEK-293T (#CRL-11268) cell lines were purchased from American Type Culture Collection (ATCC). The MC-38 (#SCC172) murine colon adenocarcinoma cell line was purchased from Sigma-Aldrich. Immortalized

	stromal vascular cells (imSVC) were generated by transducing simian virus 40 large T antigen into inguinal white adipose tissue stromal vascular fraction from 6 weeks old male mouse. Cells were maintained in DMEM (Gibco) supplemented with 10% fetal bovine serum (FBS, Gibco) and 1% pen/strep (Gibco), and incubated at 37°C in 5% CO_2.
Authentication	We ensure that each cell line has been authenticated.
Mycoplasma contamination	We did not perform a mycoplasma contamination test.
Commonly misidentified lines (See ICLAC register)	None of the cell lines used in the study were listed among the commonly misidentified lines.

Animals and other research organisms

Policy information about studies involving animals; ARRIVE guidelines recommended for reporting animal research, and Sex and Gender in Research

Laboratory animals	Adipocyte-specific BECN1 deficient mice (BaKO) were generated as described previously 20. Mice with floxed alleles of Yap1 (Yap1tm1a(KOMP)Mbp), Taz (Wwtr1tm1.2Eno), and Atg7 (Atg7tm1Tchi RBRC02759; RIKEN) were obtained from the Knockout Mouse Project (KOMP) Repository (KOMP, Davis, California, USA). BaKO mice were crossed with YAPflox/flox (Yap1tm1a(KOMP)Mbp) and TAZflox/flox (Wwtr1tm1.2Eno) to generate adipocyte-specific BECN1/YAP/TAZ KO mice (BYTaKO). Adipocyte-specific Atg7 deficient mice were generated by crossing Adipoq-Cre (JAX: 010803) with ATG7flox/flox (Atg7tm1Tchi). The excised alleles were validated using PCR analysis of genomic DNA extracted from the tail tips of mice. The genotyping primer sequences are listed in Supplementary Table 1. Adipoq-cre, BECN1flox/flox, MMTV-PyMT (PyBA) mice were generated by crossing BaKO with MMTV-PyMT (The Jackson Laboratory, Bar Harbor, ME, USA). The integrated MMTV-PyMT sequence was validated using PCR analysis of genomic DNA extracted from tail tips of mice. Mice received either a normal chow diet (NCD) or high-fat diet (HFD) (consisting of 60 kcal %), in addition to water, ad libitum, for indicated times. Immunocompromised N2G (NOD; Prkdcem1Gmcr; Il2rgem1Gmcr) mice were obtained from Gemcro Corp (Seoul, Republic of Korea). These mice were generated from NOD with deletion of entire exons of PRKDC and IL2RG. Mice used in studies were housed in a controlled environment with a 12-h light/dark cycle. Male mice were fed either a normal chow diet (NCD) or high-fat diet (HFD) for indicated times and provided with free access to food and water.
Wild animals	All mice used in the study were inbred laboratory animals.
Reporting on sex	The assay of tumor progression using EO771 syngeneic, 4T1 xenograft, and MMTV-PyMT genetically engineered mice was only performed on female mice since mammary tumors are more relevant in female subjects. Tumor progression of MC-38 was measured only in male mice without consideration of sex-dependency.
Field-collected samples	No field collected samples were used in the study.
Ethics oversight	All animal care and experiments were performed in accordance with the guidelines of the Korean Food and Drug Administration and approved by the Institutional Animal Care and Use Committees of the Laboratory Animal Research Center at Yonsei University (permit number IACUC-A-202208-1520-01).

Note that full information on the approval of the study protocol must also be provided in the manuscript.
